# Computational and Complex Network Modeling for Analysis of Sprinter Athletes’ Performance in Track Field Tests

**DOI:** 10.3389/fphys.2018.00843

**Published:** 2018-07-06

**Authors:** Vanessa H. Pereira, Claudio A. Gobatto, Theodore G. Lewis, Luiz F. P. Ribeiro, Wladimir R. Beck, Ivan G. M. dos Reis, Filipe A. B. Sousa, Fúlvia B. Manchado-Gobatto

**Affiliations:** ^1^Laboratory of Applied Sport Physiology, School of Applied Sciences, University of Campinas, Limeira, Brazil; ^2^Center for Homeland Defense and Security, Naval Postgraduate School, Monterey, CA, United States

**Keywords:** computational science, complex network, physiology, sprinter athletes, performance

## Abstract

Sports and exercise today are popular for both amateurs and athletes. However, we continue to seek the best ways to analyze best athlete performances and develop specific tools that may help scientists and people in general to analyze athletic achievement. Standard statistics and cause-and-effect research, when applied in isolation, typically do not answer most scientific questions. The human body is a complex holistic system exchanging data during activities, as has been shown in the emerging field of network physiology. However, the literature lacks studies regarding sports performance, running, exercise, and more specifically, sprinter athletes analyzed mathematically through complex network modeling. Here, we propose complex models to jointly analyze distinct tests and variables from track sprinter athletes in an untargeted manner. Through complex propositions, we have incorporated mathematical and computational modeling to analyze anthropometric, biomechanics, and physiological interactions in running exercise conditions. Exercise testing associated with complex network and mathematical outputs make it possible to identify which responses may be critical during running. The physiological basis, aerobic, and biomechanics variables together may play a crucial role in performance. Coaches, trainers, and runners can focus on improving specific outputs that together help toward individuals’ goals. Moreover, our type of analysis can inspire the study and analysis of other complex sport scenarios.

## Introduction

Mathematics, exercise, complex networks, and physiology can be integrated to answer questions asked by both the general public and scientific experts (Noakes, 2012). The integration of sports analytics with complex networks is called complex sports analytics. This new field helps scientists build predictive models for better decision-making, highlighting the importance of complex analysis that goes beyond standard statistics (Davenport and Harris, 2007). A complex network is a mathematical representation of measurable variables as nodes and its interactions as links (a graph) (Lewis, 2009). Such representation makes it possible to regard complex network structures as a promising tool for predictive models (Bashan et al., 2012). The complex network approach has been applied to a variety of studies involving inflammatory interactions in a cell (Wherry and Kurachi, 2015), human diseases as wiring maps (Barabási et al., 2011), and brain cognition analysis (Park and Friston, 2013). In physical exercise, a few studies have demonstrated the advantages of holistic analysis as follows: metabolic biomarkers that can preview/predict exhaustion (Kastellorizios and Burgess, 2015); the role of central regulation in muscular recruitment (Froyd et al., 2016); the stressors’ effect on exercise (Lloyd et al., 2016); and cycling performance and brain function (Vitor-Costa et al., 2015). By utilizing complex networks, a few studies argue their advantages for tactical–technical decision-making processes by teams (Vaz de Melo et al., 2008; Passos et al., 2011; Fewell et al., 2012), and our recent work regarding exhaustion in treadmill tethered running (Pereira et al., 2015). Running as an exercise has become a popular activity for both athletes and amateurs to maintain health or achieve performance goals. Studies have been conducted to evaluate the role of breathing parameters in running (Cottin et al., 2007) and the importance of athletes’ genetic backgrounds for sports performance (Sessa et al., 2011). However, we cannot find significant literature utilizing complex network models to analyze running exercise performance, especially under different running conditions for sprinter athletes. The purpose of this paper is to strategically analyze runners’ performance considering several parameters involved in the process, embracing both a physiological basis and biomechanics in the tests. The main novelty here is to determine a distinct manner of analyzing data in an untargeted manner, inspired by others’ work among the first to apply network physiology to analyze physiological signals (Bashan et al., 2012; Bartsch et al., 2015; D’Agostino, 2016) and metabolites in exercise (Lee et al., 2011); moreover, we use complex networks, which have proven to be a remarkable tool.

With this goal, and for the suitable analysis of high-performance athletes, we chose the supramaximal exercise (Astorino and White, 2010). For this type of exercise, we must consider not only one type of test but a data combination from many testing exercises because performance depends on various physiological factors. In our study, a set of exercise outputs has been collected and analyzed both in (i) free running on a track, representing a similar scenario to a competition, and (ii) tethered running, reflecting professional athlete training (Harrison and Bourke, 2009; Clark et al., 2010; Alcaraz et al., 2014). We have analyzed classical aerobic and anaerobic parameters (Dekerle et al., 2003; Midgley et al., 2008; Keir et al., 2016). In high-intensity exercise, precise quantifications of more than one test represent a fundamental aspect to understand sports performance and to optimize physical preparation (Gastin, 1994; Green, 1994). In this study, professional sprinters who have performed close to world records (within 90%) participated in different running tests. Each test generated measurable outputs, which have been submitted to our complexity analysis using two complex models for free and tethered running, respectively. They include biomechanics and physiological variables (Time Limit tests); anaerobic [maximum accumulated oxygen deficit (MAOD)] data (Medbø et al., 1988; Medbo and Tabata, 1989; Medbo and Burgers, 1990), and aerobic data gathered from incremental tests. These have been combined with anthropometric data for the understanding of which physiological, anthropometric, and biomechanics variables influence performance. Our main goal is to evaluate new ways of interpreting and understanding the meaning of the exercise outputs in greater performance through complex network analysis. This novel approach integrates a considerable number of variables under a global context called complex sports analytics. In the following sections, we detail the materials and methods, including the types of tests to which our volunteers submitted, and the complex network models construction. Later, we provide the results, discussions, and conclusions considering the analysis of complex networks and their metrics.

## Materials and Methods

### Athletes’ Characterization

Ten male professional sprinters have been selected. All subjects gave verbal and written informed consent, and this study has been approved by the Research Ethics Committee of São Paulo State University, Biosciences Institute (protocol no. 3527.05062009), in accordance with the Declaration of Helsinki. A free and informed consent form was signed by each participant and contains information about procedures, voluntary participation, consent to the use of data and information for further scientific publications, and certifies the non-use of any illegal substances. All experiments were performed in accordance with relevant guidelines and regulations. The participants were instructed to observe a light diet and good hydration habits and have their last meal between 2 and 3 h before testing and not to consume beverages containing alcohol at least 24 h before testing; additionally, they were requested to not practice strenuous exercises or use medications during the experimental period. Two selected individuals had to be excluded from our sample for not meeting the research criteria: lesions under treatment involving medications use, and unsatisfactory observance of evaluation tests. Thus, eight individuals (mean age, weight, height, and fat percentage of 21 ± 3 years, 71.49 ± 5.99 kg, 179.8 ± 6.15 cm, and 4.9 ± 1.27%, respectively) met all research criteria, being professional sprinters. This number of individuals analyzed can be viewed as a limitation of our work. On the other hand, we were able to analyze a significant quantity of parameters, as explained later. All participants performed tests in free and tethered running conditions. Our athletes were carefully chosen to provide relevant data. We believe our sample does represent the performance behavior of elite athletes, who compared to other athletes of the same discipline, show a mean relationship between Personal and World Records of 90.92%, as shown in **Table 1**.

**Table 1:** Athletes evaluated in our research (identified by number to protect their identity), discipline, personal record in official competitions (personal *R*), current world record (world *R*), relationship between personal and world record (%WR), record-holding athlete’s name (current WR athlete), and competition occasion with year (competition).

Athlete	Discipline	Personal *R*^∗^ (s)	World *R*^#^ (s)	%WR	Current WR athlete^#^	Competition^#^
1	Men’s 400 m	48.05	43.03	89.55%	Wayde Van Niekerk	Rio 2016
2	Men’s 400 m	46.22	43.03	93.10%	Wayde Van Niekerk	Rio 2016
3	Men’s 400 m	46.64	43.03	92.26%	Wayde Van Niekerk	Rio 2016
4	Men’s 100 m	10.50	9.58	91.24%	Usain Bolt	Berlin 2009
5	Men’s 100 m	10.40	9.58	92.12%	Usain Bolt	Berlin 2009
6	Men’s 100 m	10.84	9.58	88.38%	Usain Bolt	Berlin 2009
7	Men’s 400 m hurdles	51.75	46.78	90.40%	Kevin Young	Barcelona 1992
8	Men’s 110 m hurdles	14.17	12.80	90.33%	Aries Merritt	Bruxelles 2012
	Athletes’ mean %WR:			90.92%		


*^∗^Personal R, personal records in official competitions (seconds), available at: http://www.cbat.org.br/resultados; http://www.all-athletics.com/pt. ^#^World records for each category and competition, available at: http://www.alltime-athletics.com/; https://www.iaaf.org/; http://www.sports-reference.com/.*

### Time Limit Running Tests

Six experimental sessions were performed within a 48- to 72-h interval on a synthetic 400 m running track. The sessions consisted of an incremental test and two supramaximal bouts (110 and 120% of peak P vVO_2*max*_) for free and tethered running, all being conducted to athletes’ exhaustion. The tests were performed at approximately the same time, in daylight, for each subject, with ambient temperatures between 25 and 32°C. The VO_2_, carbon dioxide production (VCO_2_), and ventilation were monitored breath by breath using a calibrated portable gas analyzer (K4b2, Cosmed, Rome, Italy), and calibration was performed according to the manufacturer’s recommendations. In addition, heart rate (HR) was measured during tests using a transmitter belt (T61 Polar Electro, Kempele, Finland). The data for the above parameters were continuously transferred via telemetry-specific software (K4b2 Data Management Software, version 9.1b, Cosmed, Rome, Italy) and later analyzed in Excel (Microsoft Office Excel 2007 for Windows, Microsoft, United States) using the average value of 15 successive breaths (Robergs et al., 2010). Blood samples of 25 μL were collected from the earlobe of the volunteers in heparinized capillary tubes at 1, 3, 5, 7, and 9 min after testing and transferred to plastic tubes containing 400 μL of trichloroacetic acid solution at 4%, stored on ice for further lactate analysis in a microplate reader (ASYS Expert Plus UV, Biochrom, United Kingdom), applying the enzymatic method. Peak lactate has been considered for our models analysis. From both free and tethered running testing, we retained for analysis and modeling: mean velocity (km/h), peak lactate (mmol/L), peak VO_2_ (mL/min), mean VO_2_ (mL/min), HR (bpm), mean *R*, peak *R*, mean VCO_2_ (mL/min), and peak VCO_2_ (mL/min). In tethered running, we have also included in the model: power (W), force (N), work (J), step frequency (s^-1^), and step length (m). After being equipped and monitored for 5 min standing at rest to obtain baseline measurements, the volunteers warmed up by jogging for 800 m (two laps on the track), followed by 5 min of stretching. The instrument utilized in tethered running was developed by our research group (Sousa et al., 2015) and adapted from previous work (Lima et al., 2011). It consists of tricycle metal chassis with vertical shaft attached to its front portion, rubber tires and mechanical brake system, adjustable drive, front rear wheels equipped with a magnetic sensor (55110, Hamlin, United States) and load cell (CSL/ZL-250, MK, Brazil) attached to the front shaft, and can be adjusted for height. A welded basket to the chassis center contains a signal acquisition system comprises a universal DC amplifier (Gould, United States), signal conditioner (USB-6008, National Instruments, United States), and notebook system, powered by 12-V battery attached to the posterior-inferior region of the chassis. For the tethered running testing, runners were tied by the waist to the apparatus by means of a nylon belt, which was attached to the load cell using a 1.5-m steel cord. In this condition, resistances equivalent to 4% of body weight were imposed on the athletes. The load cell signals were amplified and sampled at 1000 Hz by a LabVIEW analyzer (LabView Signal Express 2009, National Instruments, United States) and subsequently analyzed using specific routines in MATLAB (MATLAB, R2008a, The MathWorks, United States).

### Incremental Tests for Aerobic Power and Capacity Determination

We utilized incremental tests in both running conditions (free and tethered) to define the exercise intensities and variables here analyzed. Incremental tests began at 9 km/h, with increased velocity of 1 km/h every 2 min until volitional exhaustion or the athlete could not support the predetermined velocity, indicated by not reaching two successive marks in the required intervals, despite intense verbal stimulus. From these tests, we determined for both tethered and free running: peak P vVO_2_ (km/h), peak PVO_2_ (mL/min), aerobic capacity (km/h), CVO_2_ (mL/min), and % peak VO_2_. The aerobic capacity was determined by the analysis of the increase of ventilator equivalents of O_2_ and CO_2_ for the exercise intensities (Rausch et al., 1991). Although the testing order has been necessarily met in each condition, the volunteers were randomly assigned. At all times, velocity was controlled by sound signals provided by an experienced evaluator, with the purpose of guiding the athletes to pass through marks placed every 50 m on the track.

### MAOD – Anaerobic Capacity Test

Supramaximal races were performed to determine the parameters necessary to calculate the MAOD, which was used in an Anaerobic Capacity test. In preparation, the volunteers performed one to two short runs (∼5–8 s). The reached velocity of each athlete was equivalent to 120 and 110% of peak P vVO_2_ achieved in the incremental tests during free and tethered running, respectively. The peak values of physiological responses achieved in these evaluations were also measured for comparison purposes. For our complex models, we utilized the MAOD (mL) parameter from this test. Statistical analysis was performed using the Microsoft Office Excel 2007 software (Microsoft, United States) and Statistica 7.0 (STATSOFT, United States).

### Complex Network Models

We have chosen to build two complex network models to understand the scenarios of performance during free and tethered running. The following variables have been defined for both models:

• Time Limit tests: mean velocity (Km/h), peak lactate (mmol/L), peak VO_2_ (mL/min), mean VO_2_ (mL/min), HR (bpm), mean R, peak R, mean VCO_2_ (mL/min), and peak VCO_2_ (mL/min)
• MAOD – Anaerobic Capacity test: MAOD (mL)
• Aerobic Power test, Peak P vVO_2_ (km/h), and Peak P VO_2_ (mL/min)
• Aerobic Capacity test: aerobic capacity (km/h), C VO_2_ (mL/min), C %VO_2_ Peak
• Anthropometric data: %Lean mass and BMI (kg/m^2^)

For tethered running, we also measured in the Time Limit test: Power (W), Force (N), Work (J), Step Freq. (s^-1^), and Step Length (m). The variables are representations of distinct levels of changes in the exercise: aerobic, anaerobic, physiological, biomechanics, and anthropometric variations. Following our previous methodology of building networks (Pereira et al., 2015) and for comparative analysis, we developed an algorithm in the Java language (IDE Eclipse, Kepler version) for calculation and analysis of Pearson correlations between two variables of the choices mentioned above. The scattering of points or quality/goodness of fit was observed, and we also analyzed the Spearman’s, Newman-Keuls and Pearsons correlations. The latter proved the most appropriate fit in both free and tethered running. The returned Pearson correlation results (c) were analyzed by another routine, which classified them as weak (-0.3 < c < 0.3), moderate (-0.7 < c = -0.3 or 0.3 = c < 0.7), or high (c = -0.7 or c ≥ 0.7). Using the console application in Eclipse, we built a function which returns the type of running test (Free or Tethered), the two variables compared by the correlation (Ex. Velocity vs. Lactate), and the final value of correlation in double precision (Ex. 0.877820878967058). Also, we determined and displayed the classification of this correlation (Ex. High). A High or Moderate correlation has been selected to indicate a link between variables that have been converted to nodes to build the networks. Our objective to build a meaningful graph leads us to utilize only high and moderate correlation results. Some previous studies have considered systems as coupled by non-linear feedback or feedforward loops; however, they utilize a distinct type of correlation calculation (Bashan et al., 2012; Bartsch et al., 2015; Liu et al., 2015). Utilizing Pearsons correlations, we considered our links starting with 0.3, a very low value, as an adequate weight for the complex models. The actual link weights in every complex model were directly influenced by the network structure and complex metrics utilized in the results. Eighteen variables became 18 nodes in the free running network from more than 100 total correlations evaluated. Twenty-three variables became 23 nodes in the tethered running network from more than 200 correlations evaluated. Each node and link have been added to another Java program, which acts as the network interface.

Once the values and the parameters are calculated and analyzed, we build a complex network for each performance scenario. Each network is a graph *G* = {*N, L, f* }, where *N* = {*n*_1_, *n*_2_,… *n_k_*} nodes, *L* = {*l*_1_, *l*_2_,… *l_m_*} links, and the mapping function is *f* : *N* x *N.* The connection matrix *C* is expressed by *f* and defines the network topology.

A node is a measurable parameter. An influence is represented by a link – node *X* is linked to node *Y* if *X* has an influence on *Y*, represented as *X* →*Y*. The influence of node *X* on node *Y* is measured as the calculated correlation coefficient of the *X* →*Y* link. Correlations have been normalized by dividing them by the maximum correlation value over all links present in the graph/network. A connection matrix is defined as *C* and is an *N* × *N* matrix of *m* links connecting all nodes. *C*_ij_ =correlation result calculated between two parameters (nodes). *C* is symmetric when links are bidirectional, e.g., *i*↔*j*. Then *C*_ij_ = *C*_ji_. if *C* is non-singular, its eigenvector is *V* = {*v*_1_, *v*_2_, …, *v_k_*}, where *v*_i_ are eigenvalues corresponding to nodes *n*_i_. The solution to [*C* -*VI*] = 0, where *I* is the identity matrix, yields the eigenvalues *V*. The degree of a node is the count of its connecting links. For eigenvalues, we have built a function Power Method (Lewis, 2009; Ray, 2016) algorithm; the pseudo code is:

*Choose a random vector* q^(0)^𝜖R*^n^*
*for k* = 1,2,… *while* || *q*^(*k*-1)^-*q*^(*k*-2)^||>*𝜖*
  *z*^(*k*)^ = *Aq*^(*k*-1)^
  *q*^(*k*)^ = *z*^(*k*)^/||*z*^(*k*)^||
  *λ*^(*k*)^ = [*q*^(*k*)^]*^T^Aq*^(*k*)^
end

All these measures displayed on-screen once the interface has been built. The betweenness centrality of node *X* is the number of the shortest paths passing through it, determined by counting all the shortest paths from all nodes to all other nodes. The presence of a correlation-weighted link represents moderate or high influences between nodes. Bi-directionality indicates possible influences in both directions (a node can affect or be affected by another node). Non-linearity is not represented in the links composition correlations idea, but the final network structure and the network metrics are utilized to look for scientific answers involving running performance. We chose bi-directionality for links because we cannot affirm that one node influences another in only one direction. Let influence vector *S*_(0)_ initially be defined as the initial state of node *N*, then the next states are *S*_(1)_ = *C* x *S*_(0)_, *S*_(2)_ = *C* x *C* x *S*_(0)_ = *C*^2^ x *S*_(0)_, and so on. Thus, *S*_(*t*)_ = *C*^t^ x *S*_(0)_. However, *VI* can replace *C*, because [*C–VI* ] = 0, so *S*_(t)_ = [*VI* ]^t^ x *S*_(0)._ Thus, the state of node *i*, represented by *s*_i_, is asymptotic to *s*_i_ = *v*_i_^t^ x *s*_*i*(0)__._ Evidently, *s*_i_ approaches infinity if *v_i_*>1, and approaches zero if *v_i_*<1. Thus, *v_i_* is a measure of the influence or importance of *n*_i_ to the network (Lewis, 2009; Pereira et al., 2015). Regarding the systems state and variables, there are two distinct points to note. Each network is fixed and modeled by correlations and variables, and each network does not change. These are the two models we have used. Considering each model can be represented by a transformation matrix, we calculate the eigenvalues and eigenvectors associated with this matrix, which indicate the direction in which the systems may converge. This corresponds to mathematical stability theory (Meyn, 2008), which helps to capture networks’ essential dynamics and clarifies the analysis.

We utilize an interface to visualize the network structure and assign link correlations as weights for both tethered and free running.

After construction of the two networks, we conducted simulations for three network metrics: degree, eigenvalue, and betweenness. All measures were exhibited on-screen once the interface was built. For the degree, we developed another function to calculate and show the number of links to each node of the network. We repeated the calculations various times and checked all values according to the network size. Each network was fixed and modeled by correlations and variables. We then calculated the degree of each node, highlighting the hub node as the one with the greatest number of connections (degree analysis). Moreover, we implemented the calculation of the eigenvalues and the networks’ betweenness centrality, highlighting the major value obtained from the calculations. Such metrics were crucial for understanding the role of each node in each complex network, and here our aim was to understand the roles of the nodes regarding an athlete’s running/sprinting performance, as presented in the following results and discussion sections.

## Results

Nodes were created and inserted, then links were added according to the results of the correlation, and weighted by the calculated value (Ex. velocity was connected to Lactate through a link with 87% influence). The influence is considered mutual, in both nodes’ directions.

**FIGURE 1:**
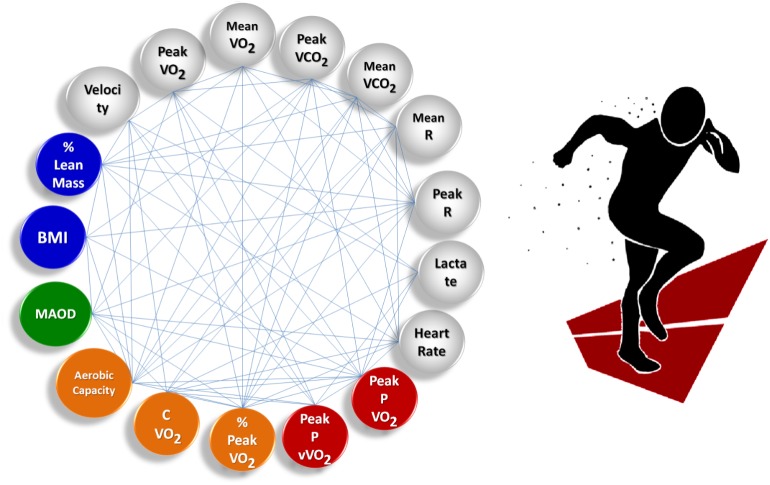
Complex sports model showing the group’s averaged complex network built for free running. All nodes in the complex network were measured and calculated for each individual. Links are assigned weights equal to the correlation coefficient between node pairs. Nodes represent anthropometric data (blue); Time Limit test data (gray); MAOD – anaerobic capacity data (green); incremental tests – aerobic capacity data (orange); and aerobic power data (red).

**Figure 1** shows the athlete group’s averaged complex network built during free running. This complex model is the result of measured and calculated parameters according to the test realized, as mentioned in the Materials and Methods section: (i) Time Limit tests, (ii) MAOD – Anaerobic Capacity tests, (iii) Incremental tests to determine aerobic power and aerobic capacity parameters and data, and (iv) Anthropometric data.

**FIGURE 2:**
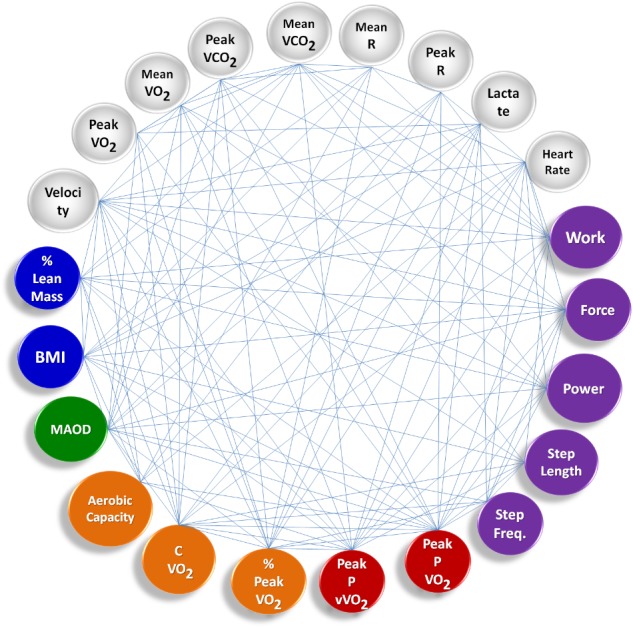
Complex sports model showing the group’s averaged complex network built during tethered running. The main difference compared to the free running scenario is that there are new nodes defined by the tethered system in the Time Limit test, representing biomechanics outputs (purple). Remaining nodes correspond to anthropometric data (blue), Time Limit test data (gray), MAOD – anaerobic capacity data (green), incremental tests, aerobic capacity data (orange), and aerobic power data (red).

**Figure 2** shows the group’s averaged complex network built during tethered running, including other measured variables for the Time Limit test, via instrument of resistance: Power (W), Force (N), Work (J), Step Freq. (s^-1^), and Step Length (m).

**Table 2** shows the statistical outcomes of each parameter measured in both types of running tests. Each parameter has been defined as a node in both network models.

**Table 2:** Parameters utilized as nodes and their first, median, and third quartile values in each model for free and tethered running tests.

Source	Variable	Free			Tethered		
			
		Q1	Median	Q3	Q1	Median	Q3
Time Limit test	Mean velocity^a^ (Km/h)	19.2	19.6	20.8	15.6	15.8	16.0
	Peak lactate (mmol/L)	9.7	10.7	11.9	9.1	9.6	10.8
	Peak VO_2_ (mL/min)	3372.5	3499.5	3550.9	3350.2	3610.5	3685.6
	Mean VO_2_ (mL/min)	2889.1	3018.6	3239.4	2989.7	3124.1	3272.1
	Heart rate (bpm)	188	191	193	173	182	186
	Mean *R*^a^	1.1	1.1	1.2	1.2	1.2	1.2
	Peak *R*	1.4	1.4	1.5	1.4	1.4	1.5
	Mean VCO_2_ (mL/min)	3528.2	3714.6	3818.0	3777.6	3930.2	4059.5
	Peak VCO_2_ (mL/min)	4826.0	4939.6	5192.9	5003.5	5305.1	5494.2
	Time limit (min)	2.3	2.7	2.9	3.0	3.4	3.8
Anthropometric data	%Lean mass	94.1	95.0	96.0	94.1	95.0	96.0
	BMI (kg/m^2^)	20.8	22.7	23.5	20.8	22.7	23.5
Aerobic power data	Peak P vVO_2_^b^ (km/h)	16.0	16.3	17.3	14.1	14.3	14.5
	Peak P VO_2_ (mL/min)	3284.1	3337.9	3818.8	3379.0	3560.7	3738.0
Aerobic capacity data	Aerobic capacity^c^ (km/h)	12.4	12.6	12.7	10.9	11.0	11.6
	C VO_2_ (mL/min)	2624.0	2715.6	2955.1	2656.4	2849.6	3099.5
	%Peak VO_2_	74.5	78.9	81.5	80.4	82.6	82.9
Anaerobic capacity data	MAOD (mL)	2699.3	3482.5	4866.7	3148.7	3425.3	4052.5


*We show data measured directly from the time limit main tests, from incremental tests – aerobic power and capacity data – and from the MAOD – anaerobic capacity data. ^*a*^Substantial differences between parameters in Time Limit tests comparing two types of sprints; ^*b,c*^Substantial difference in incremental tests, from ^b^Aerobic Power data and ^*c*^Aerobic Capacity data, calculated and analyzed (One-way ANOVA followed by Wald–Wolfowitz Test, *p* < 0.05).*

**Table 3** shows the variables that could be measured during tethered running, thanks to the tethered system linked to individuals, which produces the same running movement owing to its supporting wheels.

**Table 3:** Parameters utilized as extra nodes during tethered running.

	Power (W)	Force (N)	Work (J)	Step frequency (s^-1^)	Step length (m)
Mean	111.8	25.1	22178.0	2.9	1.5
SD	28.2	6.7	3058.3	0.1	0.1
Q1	96.8	21.6	19756.2	2.8	1.5
Median	101.0	23.0	22137.6	2.9	1.6
Q3	111.2	24.9	24766.3	2.9	1.6


*Mean values, standard deviations, first, median, and third quartile values are shown.*

**FIGURE 3:**
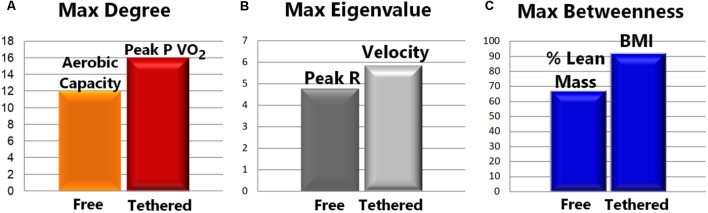
Network metrics results according to each model. **(A)** The Max Degree node (most popular node) was Aerobic Capacity in free running and Peak P VO_2_ during tethered running (both from aerobic data). **(B)** The Peak R-value (O_2_ and CO_2_ consumption tax) was the Max Eigenvalue during free running, and velocity was the maximum during tethered running (both from Time Limit tests). **(C)** Interestingly, in both models the betweenness measure points to an anthropometric measure: % Lean Mass, during free running, and body mass index (BMI), during tethered running, indicates, which node is on the central path of information flow in each network.

**FIGURE 4:**
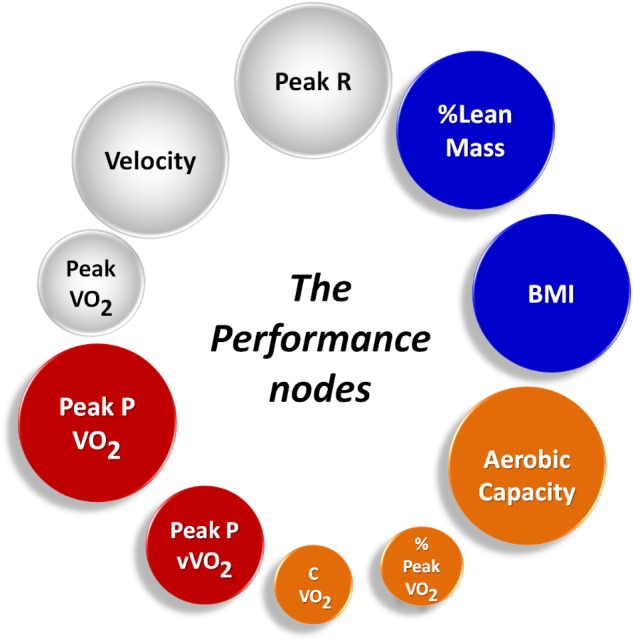
Performance nodes; the top 10 nodes weighted by their ranking positions for both running scenarios. It is possible to see: the important roles of aerobic power and capacity; the importance of anthropometric measures on the respective condition of athletes; and respiratory related data and velocity are of key importance for exercise maintenance.

Once the networks were built, it was possible to calculate topological metrics and interpret their meanings for each model, as shown in **Figures 3** and **4**. **Figure 4** illustrates each type of running and each metric analyzed, where the larger nodes have the greater weights. Thus, larger the nodes are the greater their contributions in the analysis of the performance of athletes in the analyzed scenarios.

## Discussion

The primary difficulty in today’s research is to find an effective way to interpret data. The common statistical techniques may not be able, when applied in isolation, to explore the amount and complexity of data. In this case, computation and mathematical models help us to show and interpret data, especially in sciences that do not traditionally use complex tools. Network metrics can assist in understanding the complexities in terms of parameters and interactions.

### Degree Results

The Max Degree node (most connected node) is aerobic capacity in the free running model. This indicates that when running on the track and considering the physiological basis involved, Aerobic Capacity has the greatest influence in a typical running scenario above the VO_2peak_, as it is usual in competition. The Peak P VO_2_ highlights its influence in the tethered scenario. Previously, researchers have found that Peak VO_2_ increases as a result of training and exercise (Pierce et al., 1990; Saunders et al., 2004), and that this important physiological variable is performance determinant-dependent, as shown previously (McLaughlin et al., 2010). The cardio-respiratory ability to deliver oxygen to muscles during the exercise process is demonstrated to have a critical role in our analysis, in agreement with other research (Bassett and Howley, 2000). However, here we have combined distinct tests and variables, comparing parameters and revealing the most important ones by complex metrics. In training, with the objective of improving performance, data from incremental tests (Aerobic Power test and Aerobic Capacity test) show significant degrees of influence compared to other tests jointly analyzed. These physiological-related nodes can be viewed as *hubs* in each structured network of influences, which means that Aerobic Power and Aerobic Capacity individualized evaluations are necessary for specialized athletes to sustain an ideal power intensity output for track competitions under the exercise conditions investigated.

### Eigenvalue Results

The eigenvalue of the complex system reached its maximum for Peak R (Respiratory Exchange Ratio) during free running. A high *R*-value represented here confirms the high intensity of the exercises proposed, and we interpret it as carbohydrate predominant consumption. Thus, we associate it with carbohydrate depletion, which influences the individuals’ capacity to sustain free running. This result, extracted by our analysis, is related to other investigations such as the importance of R as a fitness indicator, even in untrained individuals (Ramos-Jiménez et al., 2008), the metabolic role of R during exercise in trained athletes (Goedecke et al., 2000), and carbohydrates’ role in helping athletes to improve performance (Egan and D’Agostino, 2016; Luden et al., 2016). During tethered running, the eigenvalue metric reached its maximum for velocity in the Time Limit test. This may indicate that the velocity developed, weighted by correlations to other parameters, and has a critical role in tethered running. These results reveal a possible strategic training goal for athletes using tethered training – velocity maintenance and improvement are paramount to performance (Petrakos et al., 2016).

### Betweenness Results

Evaluating the betweenness measure in both running conditions, we found anthropometric measures for maximum values for BMI and % Lean Mass. Anthropometric measures, studied in a recent review for their potential impact on performance (Mooses and Hackney, 2016), reflect the central path of information flux in each network model. We interpret such data as having central importance when considering communications between nodes for improved performance output. The relationships weight and height, when athletes ran tethered, and % Lean Mass, when running free, indicate that these central nodes keep the entire system working and develop each athlete’s performance. Thinking of muscular mass as more energetic expenditure (and higher O_2_ consumption), the ideal relationship between height and weight (BMI) can produce greater physical aspects, such as step size and frequency during exercise. This may explain why anthropometric nodes are more central in runners. New studies should consider ideal somatotypes that fit each sport condition (Bale et al., 1986), which may help performances to reach new extremes (Thompson, 2012). Such results highlight the importance of some neglected parameters that can be taken into consideration for strategic training goals in practice. In the current tests, the individuals developed lower force, power, and work compared with our previous work (Pereira et al., 2015). But, on the athletics track, their velocity was greater. Considering the specificity of athletes’ condition is different from the average athlete, and the BMI and Lean Mass parameters assist in understanding the logical relationships involved in the capacity to maintain efforts for best physical condition in athletes. Thus, our intention was to represent elite athletes who perform at higher levels in competitions, which is why we chose the athletes carefully. The tests were complex owing to the large number of variables and results being obtained directly from athletes running on a track. This manuscript falls well within the scope and increases the knowledge base of the new emerging field of network physiology and network medicine (Ivanov et al., 2016).

## Conclusion

In running, aerobic evaluations, which highlight Aerobic Capacity and the Peak VO_2_, are structurally significant in our mathematical models to remain connected and they exert influence over oscillations of other variable outputs. Such variables are affected by important variations received from other variables because of the connections with influences in both directions. This reinforces the importance of the individual aerobic evaluation of athletes to better understand their performance. The respiratory exchange ratio (R) and the velocity in the Time Limit tests determine the weights of the influences on the performance outputs of numerous variables. This suggests paying special attention to Time Limit tests to monitor such variables in training, with a focus on R and velocity. Additionally, the entire complex system will deliver an ideal performance output with central dependence on anthropometric measures, which suggests special attention should be placed on somatotype selection for high-level performance runners (% lean mass and BMI).

The tools and computational methods utilized here highlight knowledge associations that can be applied to develop better strategies regarding running that are valid for runners and could be applicable to other sports. Complex mathematical models help in the abstraction of data, evaluation/development of metrics, and determining semantic meaning. Optimal individual conditions include anthropometric, physiological, and biomechanics outputs to improve athlete performance, all of which are emphasized in the sports models proposed in this article. Future research may take into consideration other physical, biological, tactical–technical, or psychological preparations based on the latest science advances, and other models can be built for specific exercises and/or sports. The final goal is to determine strategic methods to achieve optimal efficiency in training, exercise, and sports.

## Ethics Statement

This study was carried out in accordance with the recommendations of Research Ethics Committee of São Paulo State University, Biosciences Institute (protocol no. 3527.05062009). The protocol was approved by the Research Ethics Committee of São Paulo State University. All subjects gave written informed consent in accordance with the Declaration of Helsinki.

## Author Contributions

VP, TL, CG, and FM-G contributed to propose models ideas, wrote the main manuscript text, interpreted data, created the models, and prepared figures. LR, WB, IdR, and FS collected data from field tests and analyzed them. TL and VP developed the network software. VP and TL designed and built the complex models and made metrics. All authors reviewed the manuscript.

## Conflict of Interest Statement

The authors declare that the research was conducted in the absence of any commercial or financial relationships that could be construed as a potential conflict of interest.
